# Hemoperitoneum Secondary to Spontaneous Hepatic Artery Hemorrhage: A Case Report

**DOI:** 10.7759/cureus.78371

**Published:** 2025-02-02

**Authors:** Musab Mohmed, Amir Saber, Shaikh Sayeed Iqbal, Rafay Gul, Ali Yammahi

**Affiliations:** 1 General Surgery, Dubai Academic Health Corporation, Dubai, ARE; 2 Radiology, Dubai Academic Health Corporation, Dubai, ARE; 3 Interventional Radiology, Dubai Academic Health Corporation, Dubai, ARE

**Keywords:** hepatic artery aneurysm, hepatic artery pseudoaneurysm, spontaneous hemoperitoneum, spontaneous hepatic hemorrhage, transarterial embolization, visceral artery aneurysm

## Abstract

Spontaneous hepatic hemorrhage (SHH) is a rare condition that usually occurs secondary to a neoplastic process such as hepatocellular carcinoma. We present the case of a 71-year-old male with no history of trauma or malignancy presenting to our facility with a complaint of abdominal pain. Computed tomography scans showed hemoperitoneum, a suspected aneurysmal dilatation of a branch of the left hepatic artery, and contrast extravasation from the artery. Urgent angiography and embolization of the bleeding vessels were done successfully. Herein, we review the case as well as the relevant literature on spontaneous hepatic hemorrhage and visceral artery aneurysms.

## Introduction

Spontaneous hepatic hemorrhage is a rare surgical emergency that is most often caused by liver tumors such as hepatocellular carcinoma and hepatic adenoma. Clinical presentation is often non-specific, and diagnosis is usually established by a computed tomography (CT) scan. In hemodynamically stable patients, trans-arterial embolization represents an appropriate treatment modality [1]. We present a case of spontaneous hepatic artery hemorrhage, likely secondary to a ruptured hepatic artery aneurysm or pseudoaneurysm, successfully managed using angioembolization.

## Case presentation

A 71-year-old male presented to our emergency department with a complaint of a three-day history of generalized abdominal pain, fever, reduced physical activity, and malaise. Past medical history was only significant for asthma. He denied any past surgical history or any recent trauma. Upon arrival at the emergency department, he was alert and oriented, not in any distress, and hemodynamically normal. Hemoglobin level on arrival was 12.7 g/dL. Shortly after the initial assessment, the patient’s clinical condition deteriorated significantly. He started sweating profusely and complaining of dizziness and headache. On examination, the patient was restless, hypotensive, and tachycardic. A second intravenous line was inserted, and fluid resuscitation was initiated.

**Figure 1:**
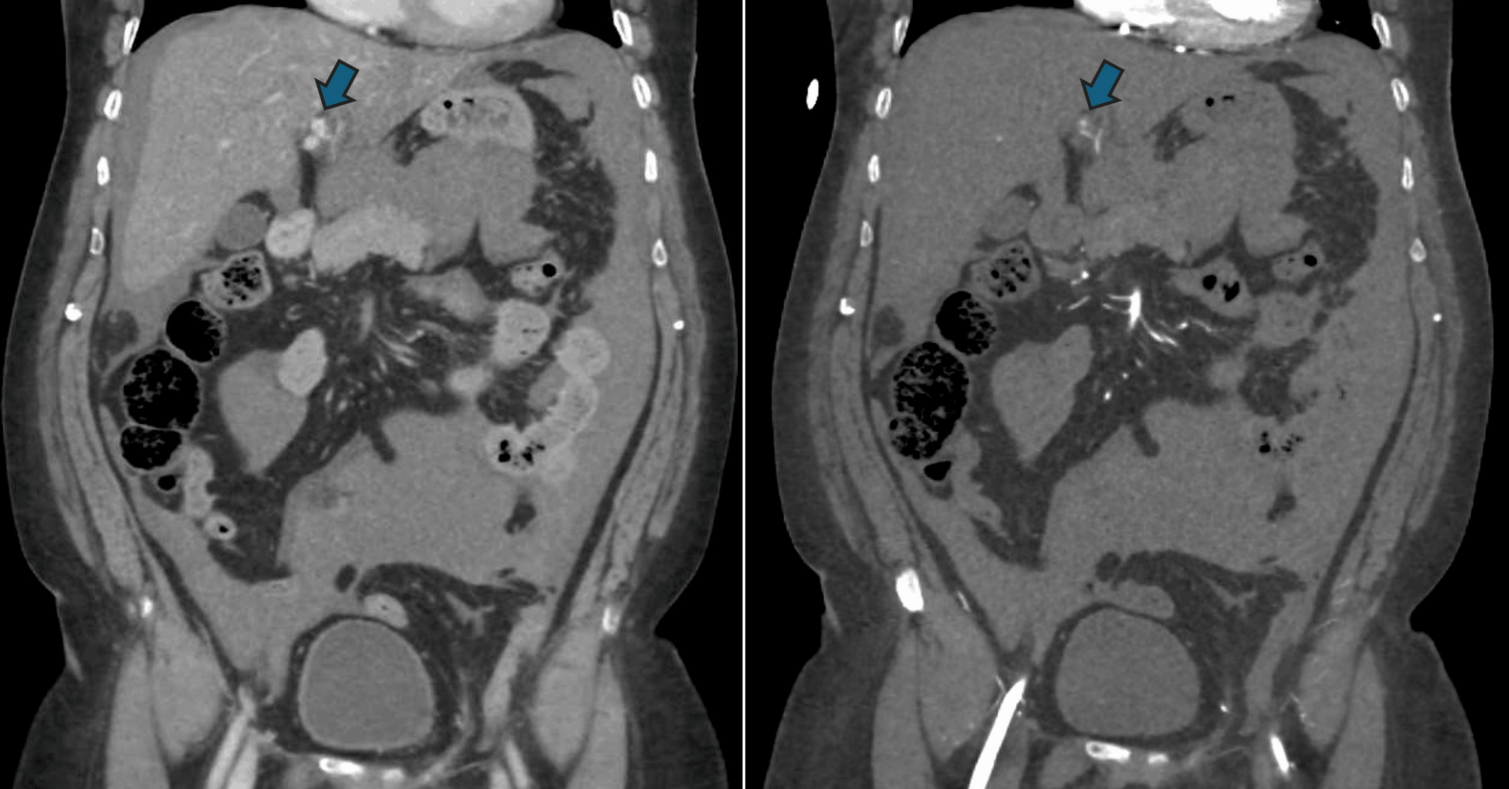
CT scans in the portal venous phase (left) and the arterial phase (right) showing hemoperitoneum and abnormal dilatation of a branch of the left hepatic artery with contrast extravasation (arrows).

After stabilization, the patient urgently underwent a CT scan of the brain as well as a CT chest and abdomen aortogram (in view of an initial suspicion of aortic dissection). He was then brought back to the resuscitation room and intubated, a central venous catheter (CVC) line was inserted, and noradrenaline infusion was started. CT scans were significant for hemoperitoneum and abnormal dilatation of a branch of the left hepatic artery with contrast extravasation (Figure 1).

**Figure 2:**
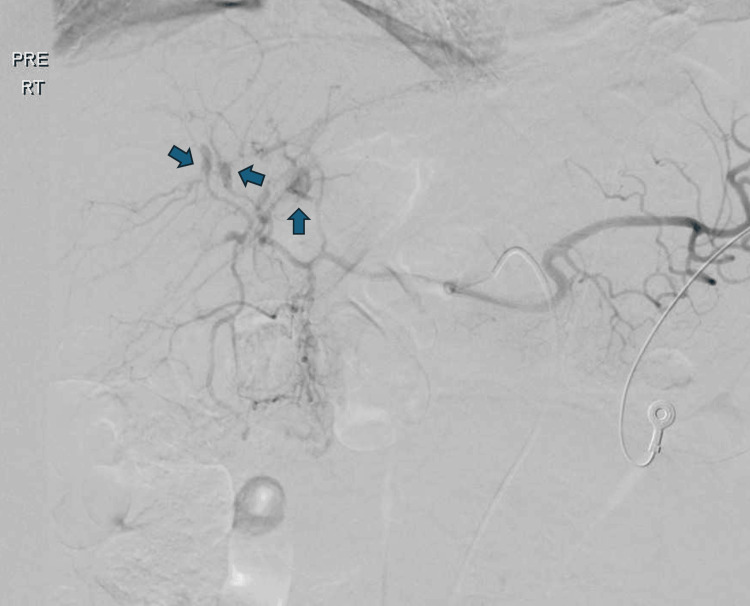
Selective celiac angiography showing active arterial bleeding from three segmental branches of bilateral hepatic arteries (arrows).

**Figure 3:**
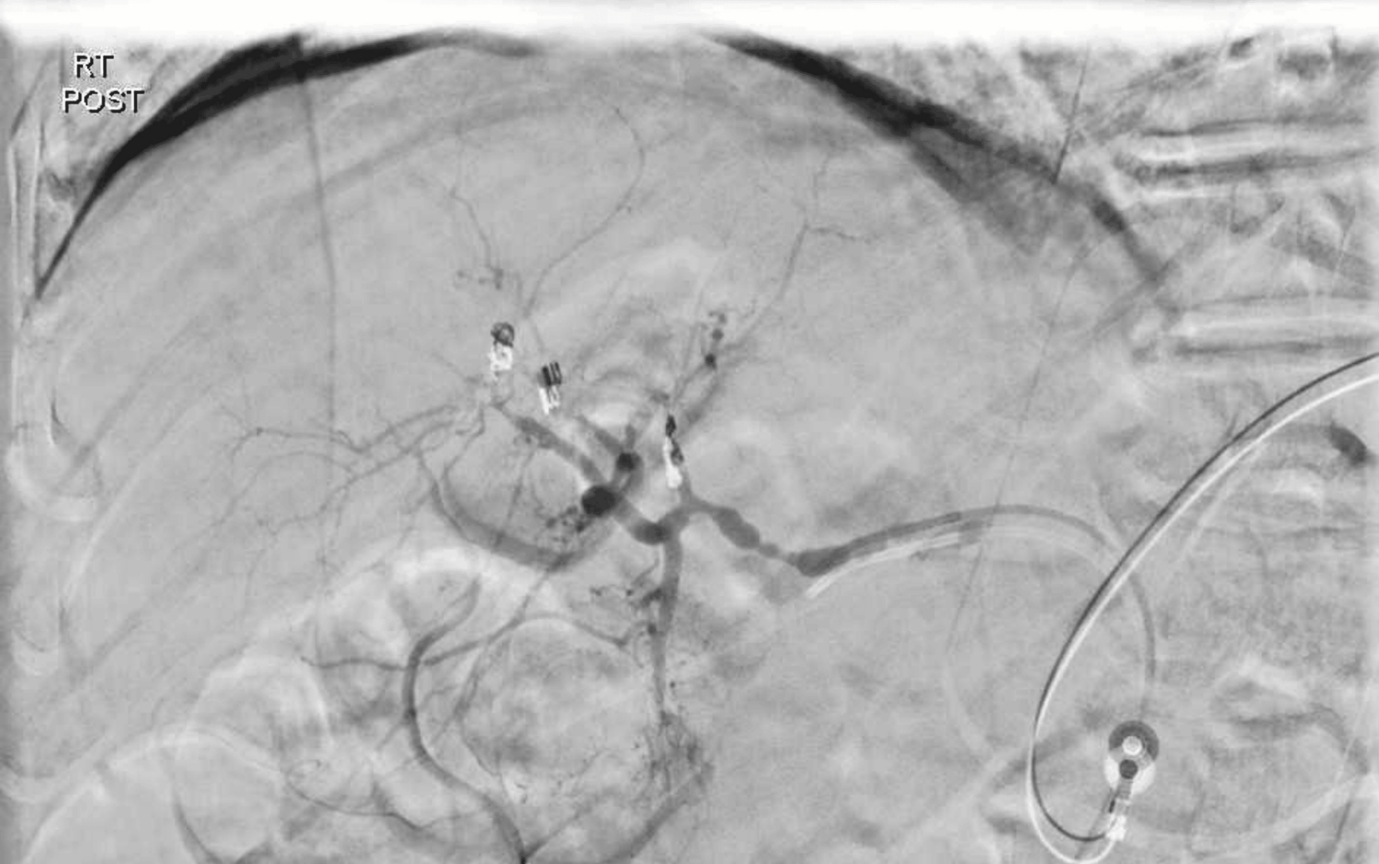
Angiogram post-embolization of bleeding hepatic arteries showing no further contrast extravasation.

Urgent selective celiac catheter angiography was done, which showed active arterial bleeding from three segmental branches of bilateral hepatic arteries (Figure 2), which were embolized using micro-coils (Figure 3).

Post-procedure, the patient was transferred to the intensive care unit. He received four units of packed red blood cells (pRBC) and four units of fresh frozen plasma (FFP) within 24 hours of the procedure. On the first day after the initial presentation, weaning from mechanical ventilation was initiated. The intensive care unit stay was complicated by an episode of sinus tachycardia on the second day, prompting a CT pulmonary angiogram, which revealed no evidence of embolism. Hemoglobin levels initially showed a gradual drop from 12.7 g/dL on presentation to 8.0 g/dL on the fifth day. After the transfusion of one more unit of packed red blood cells, the hemoglobin level improved to 9.7 g/dL and continued to increase thereafter. The patient was extubated on the sixth day and transferred out of the intensive care unit on the seventh day. By the eleventh day, the hemoglobin level had risen to 11.6 g/dL, and the patient was discharged in stable condition on the twelfth day.

## Discussion

Spontaneous hepatic hemorrhage (SHH) is a rare, life-threatening condition that requires prompt diagnosis and treatment to prevent morbidity and mortality. Hepatocellular carcinoma is the most common cause of SHH [1]. Clinical presentation is frequently non-specific, with symptoms such as abdominal pain, malaise, and vomiting being most frequently reported [2]. Initial assessment consists of obtaining a history of presenting complaints, performing a comprehensive physical examination, and blood investigations, including complete blood count, liver function tests, urea, creatinine, electrolytes, coagulation profile, and tumor markers [1].

CT scan is the diagnostic modality of choice [1]. However, in patients who are hemodynamically unstable despite resuscitation attempts and in the presence of a condition predisposing to SHH (e.g., hepatocellular carcinoma), surgical management is often undertaken through damage control laparotomy, with the diagnosis being made intraoperatively [3]. Our patient initially underwent a CT chest and abdomen aortogram due to an initial suspicion of aortic dissection. Another differential diagnosis that could have presented similarly is a ruptured abdominal aortic aneurysm (AAA). As previously mentioned, the clinical presentation in this case is vague and non-specific, highlighting the importance of imaging as soon as clinically feasible.

In the absence of any predisposing conditions, the absence of any liver masses on imaging, and in view of the dilatation of a branch of the hepatic artery with contrast extravasation on CT scan and celiac angiography, we believe that a feasible underlying etiology of our patient’s condition is a hepatic artery aneurysm (HAA). Visceral artery aneurysms (VAAs) are rare, accounting for only 5% of all intraabdominal aneurysms [4]. HAA is the second most common type of VAA after splenic artery aneurysm [5,6]. Diagnosis is usually made incidentally on a CT scan. HAAs can be classified into true aneurysms and pseudoaneurysms, with distinct clinical and radiological features. Pseudoaneurysms are often associated with a history of arterial trauma, malignancy, inflammatory conditions, or biliary procedures. Radiological features of pseudoaneurysms include focal disruption of an otherwise normal artery, as well as signs of inflammation surrounding the aneurysm [7]. True VAAs are often degenerative or secondary to atherosclerosis. Other causes of true VAAs include fibromuscular dysplasia and connective tissue disorders such as Ehlers-Danlos syndrome [4].

The Society for Vascular Surgery’s clinical practice guidelines for the management of visceral artery aneurysms recommend repair of all hepatic artery pseudoaneurysms due to the high risk of rupture and mortality. They also recommend repairing all symptomatic HAAs regardless of size and repairing asymptomatic HAAs larger than 2 cm in patients without significant comorbidities or if the rate of growth is more than 0.5 cm per year. In patients with significant comorbidities, they recommend open repair of HAAs larger than 5 cm [4].

Treatment options include surgical arterial ligation or reconstruction and endovascular embolization or stenting [4], with multiple case studies reporting the successful use of coil embolization for the treatment of ruptured hepatic artery pseudoaneurysms [8,9,10]. Endovascular treatment is minimally invasive, allows for visualization of collateral circulation, and is associated with lower mortality compared to surgical management [11].

## Conclusions

In summary, SHH is a life-threatening condition that requires urgent recognition and treatment. It often occurs secondary to malignancy, but in patients without history or radiological findings suggestive of malignancy, other etiologies, including ruptured aneurysms, need to be considered. Ruptured hepatic artery aneurysms or pseudoaneurysms require urgent treatment to prevent mortality, with an endovascular approach being a viable option if liver arterial circulation can be preserved.
